# Clinical significance of D-dimer levels in refractory *Mycoplasma pneumoniae* pneumonia

**DOI:** 10.1186/s12879-020-05700-5

**Published:** 2021-01-06

**Authors:** Xia Huang, Dan Li, Feng Liu, Deyu Zhao, Yifan Zhu, Heng Tang

**Affiliations:** Department of Respiratory Medicine, Children’s Hospital of Nanjing Medical University, Nanjing, China

**Keywords:** *Mycoplasma pneumoniae*, Refractory *Mycoplasma pneumoniae* pneumonia, D-dimer, Child

## Abstract

**Background:**

The levels of serum D-dimer (D-D) in children with *Mycoplasma pneumoniae* pneumonia (MPP) were assessed to explore the clinical significance of D-D levels in refractory MPP (RMPP).

**Method:**

A total of 430 patients with MPP were enrolled between January 2015 and December 2015 and divided into a general MPP (GMPP) group (*n* = 306) and a RMPP group (*n* = 124). Clinical data, D-D level, white blood cell (WBC) count, proportion of neutrophils (N%), C-reactive protein (CRP), erythrocyte sedimentation rate (ESR), alanine transaminase (ALT), aspartate aminotransferase (AST), and lactate dehydrogenase (LDH) were compared between the two groups. Multivariate logistic regression was performed to identify independent predictors of RMPP.

**Results:**

(1) Hospitalization time, preadmission fever duration, total fever duration, WBC, N %, CRP, LDH, ESR, ALT, AST, and D-D were significantly higher in the RMPP group than those in the GMPP group (all *P* < 0.05). (2) Correlation analysis showed that D-D was positively correlated with WBC, CRP, ESR, and LDH, and could be used to jointly evaluate the severity of the disease. (3) Multivariate logistic regression analysis identified preadmission fever duration, CRP, LDH and DD as independent risk factors for RMPP (all *P* < 0. 05). D-D had the highest predictive power for RMPP (*P* < 0.01). The D-D level also had a good ability to predict pleural effusion and liver injury (all *P* < 0.01).

**Conclusion:**

Serum D-D levels were significantly increased in patients with RMPP, indicating that excessive inflammatory response and vascular endothelial injury with prolonged duration existed in this patient population. Increased levels of serum D-D may be used as an early predictor of RMPP and the occurrence of complications. Our findings provide a theoretical basis for the early diagnosis of RMPP, early intervention and excessive inflammatory response in the pathogenesis of mycoplasma.

## Background

*Mycoplasma pneumoniae* pneumonia (MPP) is a common etiology of childhood community-acquired pneumonia (CAP), accounting for 10–40% of cases, of which, nearly 20% require hospitalization [1–3]. The clinical manifestations of MPP are complex and varied. In addition to pulmonary involvement, MPP is frequently accompanied by intrapulmonary and extrapulmonary multisystem damage. Refractory M. pneumoniae pneumonia (RMPP) has become increasingly common in recent years. RMPP frequently shows no improvement in clinical and radiological findings despite appropriate macrolide treatment and even present with necrotizing pneumonia, airway occlusion, or thrombosis [4]. The specific pathogenesis of RMPP remains unclear, and pathogenic substances or other host factors may be the cause of lung injury associated with an excessively strong immune response [5–8]. In practice, it often appears as higher levels of clinical indicators such as CRP, ESR, LDH, and other biomarkers [6–10]. Therefore, early use of immune modulators such as corticosteroid medications, is recommended in RMPP rather than waiting for antibiotic treatment to exert an effect, which could reduce M. pneumoniae (MP) -mediated immune injury and improve treatment efficacy [11–13]. However, despite the use of corticosteroids, some patients with RMPP still have persisting fever and radiological deterioration because of the formation of mucus plugs, which requires investigation using bronchoscopy [14]. There is no specificity in early clinical manifestations of RMPP, and early diagnosis is difficult; therefore, early predictors need to be identified. Several cases of MPP complicated with thrombus have recently been reported [4, 15], indicating that children with MPP have abnormal coagulation. The serum D-dimer (D-D) level can be used as a molecular marker for hypercoagulability [4], as well as an indicator for monitoring inflammation and severe infection, such as coronavirus disease 2019(Covid-19) [16]. The present study further explored the role of hypercoagulability in the pathogenesis of RMPP by evaluating D-D levels and changes in D-D levels in children with MPP.

## Methods

### Study population

Children with MPP admitted to the respiratory department of our hospital from January 2015 to December 2015 were eligible for participation in the present study. Inclusion criteria were as follows: (1) age ≥ 1 year old; (2) signs and symptoms indicative of pneumonia on admission, including fever, cough, abnormal lung auscultation, and a new infiltrate on chest radiography; (3) diagnosis of MP infection based on positive serologic test results (serum anti-MP IgM titer ≧1:160 or increased antibody titers ≧4-fold) while having positive MP polymerase chain reaction (PCR) results for nasopharyngeal secretions; exclusion of other respiratory tract infections and tuberculosis using the following tests: protein purified derivative (PPD), blood cultures, pleural effusion cultures, nasopharyngeal aspirate/swab cultures, nasopharyngeal aspirate/swab for virus antigen detection (respiratory syncytial viruses, influenza viruses, metapneumovirus, adenovirus, and parainfluenza virus), and serology for *Chlamydia pneumoniae Legionella pneumoniae*, respiratory syncytial viruses, influenza viruses, metapneumovirus, adenovirus, and parainfluenza virus.

The exclusion criteria were (1) immunodeficiency disease and (2) respiratory diseases such as primary ciliary dystrophy, cystic fibrosis, congenital bronchopulmonary dysplasia, vascular ring malformation, bronchial foreign body, asthma, pulmonary tuberculosis, pulmonary tumor and noninfectious interstitial pulmonary disease.

RMPP was defined as follows: 1) prolonged fever for 7 days or more or 2) radiological deterioration despite appropriate antibiotic treatment, including macrolides. All other participants were considered to have general M. pneumoniae pneumonia (GMPP). The parents of all participating children provided written informed consent prior to inclusion in the study.

### Data collection

All children were admitted to the hospital within 24 h after routine screening for infection, including peripheral white blood cell (WBC) count, proportion of neutrophils (N %), proportion of lymphocytes (L %), C-reactive protein (CRP), erythrocyte sedimentation rate (ESR), sputum culture, *M. pneumoniae* DNA, phlegm and blood respiratory etiology examination, levels of serum D-D, lactate dehydrogenase (LDH), alanine transaminase (ALT), aspartate aminotransferase (AST), X-ray chest radiograph (or chest CT), and electrocardiogram (ECG). General information including sex, age, history, preadmission fever duration, total fever duration, medical history, physical examination, and complications were documented, as well as an assessment of the patient’s condition. Bronchoscopy was indicated when lobar consolidation or atelectasis persisted on chest X-ray film after appropriate antibiotic and corticosteroid treatment for 1 week.

The diagnostic criterions for myocardial injury were as follows: (1) clinical manifestations of cardiac insufficiency; (2) cardiac enlargement; (3) ECG changes such as ST-T changes and arrhythmias and increased levels of CK-MB or positive cardiac troponin, excluding children with previous underlying heart disease.

The diagnostic criterions for liver injury were ALT ≥3 times the upper limit of normal (ULN). Viral hepatitis, metabolic diseases, and other diseases involving the liver were excluded.

The levels of serum D-D were determined by immunoturbidimetry using an ACLTOP700 automatic blood coagulation analyzer (Wofen Inc., USA) in accordance with the manufacturer’s instructions. The normal range of serum D-D was ≦280 ng/ml. In the present study, the degree of D-D elevation was defined as follows: mild increase: < 5-fold increase (> 280 to < 1400 ng/ml), moderate increase: < 10-fold increase (≧ 1400 to < 2800 ng/ml), and severe increase: > 10-fold increase (≧ 2800 ng/ml). Patients with significantly increased levels of D-D (≧1400 ng/ml) were reviewed after treatment for 1 week, and WBC and CRP were also reviewed at this time point.

### Statistical analysis

All statistical analyses were conducted using IBM SPSS Statistics for Windows, version. 22.0 (IBM Corp., Armonk, NY, USA). For continuous variables, comparison of means was conducted using a t-test. For categorical variables, χ2 or Fisher’s exact test was used. For ordinal scaled data, the Wilcoxon rank-sum test was used. Skewed distribution data were expressed as median values (25th–75th interquartile ranges), and comparisons between two groups were conducted using the Mann-Whitney U rank sum test. Spearman correlation analysis was used for correlation of non-normal distribution data. The Wilcoxon rank sum test was used to compare serum D-D levels before and after treatment. The critical value of the diagnostic value of each predictor was obtained using receiver operating characteristic (ROC) curve analysis. Logistic regression analysis was performed to select the variables associated with RMPP and complications. Values of *P* < 0.05 were considered statistically significant.

## Results

### Characteristics of the study subjects

**Fig. 1:**
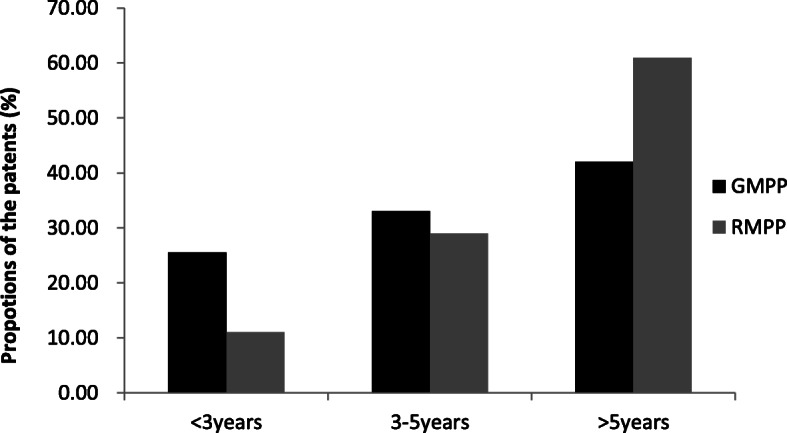
Age distribution of patients with GMPP and RMPP this study. GMPP: general *Mycoplasma pneumoniae* pneumonia; RMPP: refractory *Mycoplasma pneumoniae* pneumonia

A total of 944 patients with community-acquired pneumonia aged ≥1 year were screened for inclusion in the study. Of these, 135 patients with incomplete data were removed from further analysis, and the remaining 809 patients underwent the study assessments. Among these 809 patients, 508 (62.8%) had a positive MPP PCR result and a significant antibody response, including 78 cases of mixed infection with mycoplasma and other pathogens. After exclusion of mixed infection, a total of 430 patients with MPP were selected for further analysis including 306 GMPP cases and 124 RMPP cases. The mean age of the patients was 4.6 ± 2.5 years, with a male to female ratio of 1.23:1. Most (60.5%) of the RMPP patients were older than 5 years old, and the age group of > 5 years had the highest proportion of both GMPP and MPP cases. The age distribution is shown in Fig. 1. The RMPP group had a higher mean age than the GMPP group, with a statistically significant difference (*P* < 0.001). No difference was found in the male to female ratio. Hospitalization time, preadmission fever duration and total fever duration were longer in the RMPP group. Moreover, the levels of WBC, CRP, N %, ESR, LDH, ALT, AST and D-D were higher in the RMPP group (all *P* < 0.05; Table 1).

**Table 1:** Clinical and Laboratory data of the GMPP and RMPP cases

Characteristics	GMPP (*n* = 306)	RMPP (*n* = 124)		*P-*value
Male/female	176/130	61/63		0.116
Age in years	4.3 ± 2.5	5.7 ± 2.7		< 0.001
Hospitalization time(d)	8(7, 9)	11(9, 15)		< 0.001
Preadmission fever duration(d)	5(0, 7)	9.5(6, 12)		< 0.001
Total fever duration(d)	5(0, 7.5)	10(8, 13)		< 0.001
WBC(× 10^9^/L)	8.5 ± 3.7	10.1 ± 4.8		< 0.001
N %	54.6 ± 15.4	68.0 ± 12.8		< 0.001
L %	35.4 ± 14.4	22.6 ± 11.8		< 0.001
CRP(mg/L)	11.4 ± 15.2	40.0 ± 45.6		< 0.001
ALT(U/L)	16.1 ± 13.0	45.1 ± 63.5		< 0.001
AST(U/L)	31.9 ± 13.4	48.2 ± 59.7		< 0.001
LDH(U/L)	328.3 ± 467.4	467.4 ± 259.7		< 0.001
ESR(mm/h)	32.0 ± 15.4	44.5 ± 23.3		< 0.001
D-D(ng/ml)	247(173.0, 385.8)	1718.5(819.5, 3057.5)		< 0.001

*GMPP* general *Mycoplasma pneumoniae* pneumonia; *RMPP* refractory *Mycoplasma pneumoniae* pneumonia. *WBC* white blood cell; *N%* proportion of neutrophils; *L%* proportion of lymphocytes; *CRP* C-reactive protein; *ALT* alanine aminotransferase; *AST* aspartate aminotransferase; *LDH* lactate dehydrogenase; *ESR* erythrocyte sedimentation rate; *D-D* d-dimer

### Correlation analysis of D-D level with WBC, CRP, LDH, and ESR

WBC was normal in MPP patients, and it was increased in patients with RMPP, as well as D-D (Table 1), and the D-D level was found to be positively correlated with WBC (Spearman *r* = 0.211, *P* < 0.001). The D-D level was also found to be positively correlated with CRP, LDH, and ESR (Spearman r = 0.452, *P* < 0.001; Spearman *r* = 0.448, *P* < 0.001 and Spearman *r* = 0.376, *P* < 0.001, respectively, Table 2).

**Table 2:** Correlation analysis of serum D-D level with WBC, CRP, LDH, and ESR

	WBC	CRP	LDH	ESR
Spearman r	0.211	0.452	0.448	0.376
P	0.000	0.000	0.000	0.000

*D-D* d-dimer; *WBC* white blood cell; *CRP* C-reactive protein; *LDH* lactate dehydrogenase; *ESR* erythrocyte sedimentation rate

### Predictive value of D-D level for RMPP

**Fig. 2:**
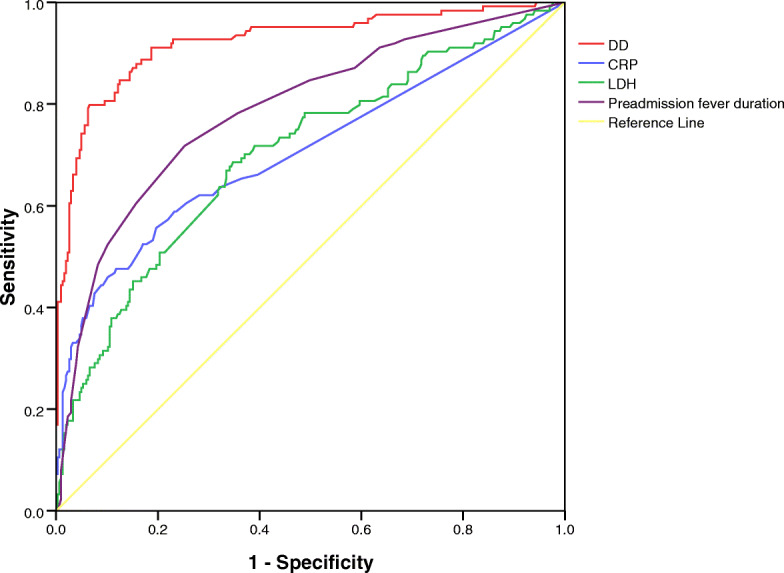
ROC curves of CRP, LDH, preadmission fever duration, and D-D for predicting RMPP. DD had better diagnostic ability for differentiation of RMPP with the best cut-off of 738 ng/ml, AUC of 0.923. ROC: Receiver operating characteristic; CRP: C-reactive protein; LDH: lactate dehydrogenase; D-D: d-dimer; RMPP: refractory *Mycoplasma pneumoniae* pneumonia; AUC: area under the curve

Univariate analysis identified 11 variables (age, preadmission fever duration, WBC, N%, L%, CRP, ALT, AST, LDH, ESR and D-D) as significant risk factors (*P* < 0.05, Table 1). The hospitalization time and total fever duration were not included because they were advanced indicators. The 11 variables were put into the multivariate regression model. Multivariate logistic regression identified preadmission fever duration, CRP, LDH and DD as independent risk factors for RMPP after adjustment for confounders (P < 0.05, Table 3). The cutoff values for preadmission fever duration, CRP, LDH and D-D were 6.5 days, 18.5 mg/L, 339 IU/L, and 738 mg/ml, respectively. D-D was found to have the highest predictive power for RMPP (*P* < 0.001, Fig. 2). For D-D levels > 738 ng/ml, the sensitivity and specificity of the prediction for liver injury were 79.8 and 93.5%, respectively.

**Table 3:** Independent risk factors for RMPP

Characteristics	OR	95%CI	P
Preadmission fever duration≧ 6.5d	1.211	1.124–1.308	< 0.001
CRP≧18.5 mg/L	1.017	1.002–1.033	< 0.05
LDH≧339 U/L	1.002	1.000–1.003	< 0.05
DD≧738 ng/ml	1.002	1.002–1.003	< 0.001

*RMPP* refractory *Mycoplasma pneumoniae* pneumonia; *CRP* C-reactive protein; *LDH* lactate dehydrogenase; *D-D* d-dimer

### Predictive value of D-D level for MPP with complications

ROC curve analysis was also performed to further evaluate the value of the D-D level in predicting complications such as atelectasis, pleural effusion, liver injury, skin rash, myocardial damage, pulmonary embolism and mucus plug formation. The results showed that the serum D-D level could robustly predict pleural effusion and liver injury (*P* < 0.001). For D-D levels > 930 ng/ml, the sensitivity and specificity of the prediction for pleural effusion were 80.6 and 60.5%, respectively. For D-D levels > 2100.5 ng/ml, the sensitivity and specificity of the prediction for liver injury were 93.3 and 72.7%, respectively.

### Degree of D-D elevation and complications

**Fig. 3:**
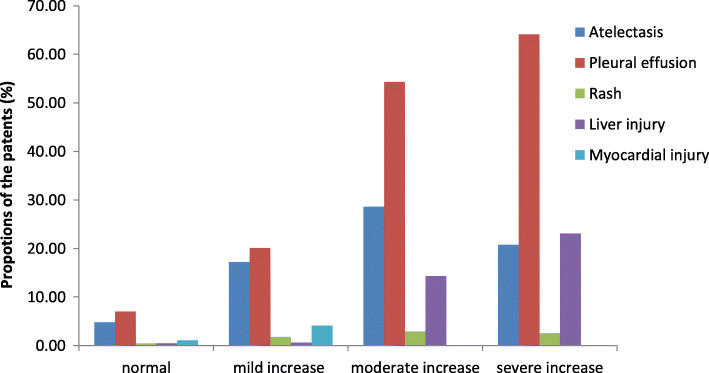
Relationship between degree of D-D elevation and prevalence of complications. The normal range of serum D-D was ≤280 ng/ml. The degree of D-D elevation was defined as follows: mild increase: < 5-fold increase (> 280 to < 1400 ng/ml), moderate increase: < 10-fold increase (≥ 1400 to < 2800 ng/ml), and severe increase: > 10-fold increase (≥ 2800 ng/ml). Pleural effusion was the most presence complication across the four classifications, The incidence of pleural effusion increased with increasing D-D levels and differed significantly among the normal, mild increase, and moderate increase groups (*P* < 0.01). D-D: d-dimer

Levels of D-D were classified as normal, mildly increased, moderately increased, and severely increased. Pleural effusion was the most presence complication across the four classifications, The incidence of pleural effusion increased with increasing D - D levels and differed significantly among the normal, mild increase, and moderate increase groups (*P* < 0.01); however, the difference between the moderate increase group and the severe increase group was not statistically significant (*P* > 0.05; Fig. 3). The incidence of atelectasis was significantly higher in the group with mildly elevated D-D levels than in the normal group (*P* < 0.01) but did not further increase with increasing D-D levels (moderately elevated group, *P* = 0.622; severely elevated group, *P* = 0.421). Only one cases of pulmonary embolism were found in our study, who was with a lower right pulmonary artery (D-D = 2401 ng/ml) and discharged without complications. She was currently in good health by follow-up.

### Serum D-D level after treatment for one week

A total of 74 children were reviewed for D-D level, WBC, and CRP after treatment for one week, including 5 patients with GMPP and 69 patients with RMPP. After treatment, the levels of WBC and CRP in most children were reduced to normal (63.0 and 81.5%, respectively). In both the GMPP and RMPP groups, the D-D level decreased significantly with treatment (*P* < 0.05, Table 4), although 91.4% of children had D-D levels that remained above the upper limit of normal (> 280 ng/ml), and 27.1% of children had a moderate-to-severe increase in D-D, all of which were within the RMPP group. According to the D-D level after a further one-week interval, patients were divided into a normal group and an abnormal group. More pleural effusions were observed in the abnormal group than in the normal group (67.2 and 28.6%, respectively, *P* = 0.04).

**Table 4:** Serum D-D levels between GMPP group and RMPP group before and after treatment

Treatment time	D-D(ng/ml)	
	GMPP (*n* = 5)	RMPP (*n* = 69)
0 day	1816.0(1368.0, 2696.5)	2984.0(2134.0, 3757.0)
1 Week z P	450.0(259.0, 633.5) −2.02 0.043	1060.0 (670.0, 2134.0) −6.45 0.000

After treatment for one week, the D-D level decreased significantly with treatment in both the GMPP and RMPP groups (P < 0.05). 91.4% of children had D-D levels that remained above the upper limit of normal (> 280 ng/ml) and 27.1% of children had a moderate-to-severe increase in D-D, all of which were within the RMPP group. *D-D* d-dimer; *GMPP* general *Mycoplasma pneumoniae* pneumonia; *RMPP* refractory *Mycoplasma pneumoniae* pneumonia

### Serum D-D level and mucus plug formation

A total of 77 MPP patients received bronchoscopy, and 28 cases (36.4%) had mucus plug formation under bronchoscopy. There was statistically significant difference in D-D level between the bronchoscope group and the non-bronchoscope group (*P* < 0.001). However, there was no statistically significant difference in D-D level between the mucous plug group and the nonmucous plug group (*P* = 0.093).

## Discussion

MPP is a common respiratory disease in children, and macrolides are the first choice antibiotics. In recent years, the prevalence of RMPP has been increasing, particularly in Asian countries [2, 6]. Clinical cases of MPP complicated with thrombosis are not unusual, and MPP complicated with deep vein thrombosis is frequently reported [4, 15]. At present, the mechanism by which MPP causes vascular embolization is not fully understood, and it is primarily considered to be related to immune mediation after vascular injury [17]. Under normal circumstances, the coagulation and fibrinolytic systems are in a dynamic equilibrium state. When coagulation occurs in vivo, thrombin acts on fibrin to activate the fibrinolytic system, and D-D can be formed [18]. D-D is primarily used in clinical settings for the initial diagnosis of pulmonary embolism, and shows diagnostic accuracy in the diagnosis of acute pulmonary embolism. Recently, D-D has been shown not only to be a special marker of the fibrinolytic system but also to be an indicator for monitoring inflammation and severe infection [16]. Levels of D-D are also closely related to the inflammatory response and may reflect the effects of infection on coagulation in infectious diseases. Some studies have reported that the levels of D-D are closely related to the severity of CAP [19].

Previous studies [20, 21] have reported that levels of D-D in children with MPP were higher than those in healthy children and were also higher in severe cases of MPP compared with mild cases, especially in severe MPP with extrapulmonary complications [21]. Few studies have reported on D-D levels in RMPP or on the monitoring of D-D levels after treatment. Consistent with previous reports, we found that the levels of D-D in the GMPP and RMPP groups were all above the normal ranges and that the levels in the RMPP group were significantly higher than those in the GMPP group. Elevated D-D levels may imply that hypercoagulability is prevalent in children with MPP and is more severe in children with RMPP and that vascular endothelial cell injury caused by an excessive inflammatory response may be involved in the mechanism of lung injury in RMPP. In a previous study, the levels of D-D were found to have decreased significantly after a period of treatment, and the final levels after treatment were higher in a group with severe pneumonia than in a group with mild pneumonia [21]. Our study found that one week of treatment was associated with a significant decrease in D-D levels, although levels remained abnormal in most cases, indicative of a prolonged state of high coagulation and endothelial injury in RMPP.

LDH and CRP, which are elevated in several pulmonary diseases, have previously been associated with RMPP and the formation of bronchial mucus plugs, and can be used as early predictors of the condition [6–8, 14]. In the present study, D-D level was positively correlated with WBC, CRP, and ESR. The increased levels of these indexes may represent a stronger systemic inflammatory response in RMPP, and possibly DD, as we found in the present study, while the positive correlation of D-D with these inflammatory indicators, which suggests that the D-D level may be used to evaluate the inflammatory response and jointly evaluate the severity of the disease. The average age of patients in the RMPP group was 5.7 years, which was older than that in the GMPP group and was consistent with previous studies [6, 7], indicating that older children have a stronger immune inflammatory response that can more readily lead to refractory conditions. A previous study showed that CRP≧50 mg/L and LDH≧480 U/L were associated with longer time to radiographic clearance [22], while ROC curve analysis revealed that the percentage of neutrophils, CRP, and LDH were useful in differentiating patients with RMPP from those with GMPP [6]. In this study, we found that CRP, LDH, and D-D levels were independent risk factors for RMPP, and after comparing the predictive value of CRP, LDH, and D-D for RMPP by ROC analysis, we found that D-D was better able to predict RMPP compared with the other indexes and may therefore be used in the early detection of refractory cases.

Regarding complications, previous studies have reported that the D-D level was positively correlated with extrapulmonary complications in pediatric patients with MPP [21]; however, extensive research on the D-D level and complications has not been reported to date. In a previous study, a higher D-D level was associated with more extensive and serious thrombosis [4]. Our results show that elevated levels of D-D had a good predictive ability for pleural effusion and liver injury and that the incidence of pleural effusion increased with increasing levels of D-D, but there was no significant difference between the moderately elevated group and the severely elevated group. The incidence of atelectasis was only significantly different between the normal D-D level group and the mildly elevated group and the incidence of rash and myocardial damage did not differ significantly between the four groups, which suggested that severely elevated D-D might not be associated with more complications. Moreover, D-D has no obvious correlation with mucus plug formation, which may be related to the hypersecretion of mucus. Therefore, further research including case studies is needed to verify this hypothesis and to avoid overtreatment.

Most of the MPP patients had higher D-D levels, but the incidence of embolic disease was lower in our and previous research [4]. It is more closely related to pleural effusion and liver function damage but not atelectasis and mucus plug formation. Therefore, elevation of D-D may be associated with other inflammatory reactions from insults of MP infection, and further research is needed. Since MPP is a self-limited disease and the host excessive inflammatory response against insults from MP infection is believed to be responsible for lung cell injury, endothelial injury and other cells, the intensity of systemic inflammation during this process is reflected in laboratory parameters such as CRP, LDH, other cytokines and chemokines, and possibly D-D. The immunopathogenesis mechanisms of acute lung injury, skin rashes, and other organ involvement in MP infection are not fully understood. The protein-homeostasis-system hypothesis in MP infection has been proposed [23]. During the incubation period of MP infection, substances including pathogenic proteins are produced in a focus. The substances spread and reach lung cells as main target cells, and various tissues for extrapulmonary manifestations via systemic circulation. Immune cells start to control these substances, not only those originating from pathogens, but also those originating from injured infected-host cells including pathogenic proteins and pathogenic peptides. Then clinical symptoms and signs such as fever, pneumonia, and occasional extrapulmonary manifestations such as skin rash, encephalopathy begin to appear, along with the involvement of other organ cells and associated elevations of AST, ALT, LDH, CRP, D-D and other biomarkers Therefore, early systemic immune modulators (dose-adjusted corticosteroids and/or high-dose intravenous immunoglobulin), may prevent rapid progression of pneumonia and induce rapid recovery of pulmonary lesions in *M. pneumoniae* pneumonia [11, 24].

Our study had some limitations. First, as this was a retrospective study, selection bias might exist, and further prospective studies are potentially needed. Second, normal control group was not established. Third, only once IgM serologic test was performed and diagnostic error may have occurred. Fourth, our study was based on a single center for data, which might have potential biases.

## Conclusion

Children with RMPP were shown to have significantly increased serum levels of D-D, indicating that severe hypercoagulability, excessive inflammatory response and vascular endothelial injury with prolonged duration may exist in this patient population. Elevated serum D-D levels may be an early predictor of RMPP, pleural effusion and liver injury. D-D detection is conducive to the early diagnosis of RMPP, early intervention of hypercoagulability and excessive inflammatory response.

## Data Availability

The raw data is available upon reasonable request from the corresponding authors.

## Abbreviations

D-D: D-dimer
MP: *Mycoplasma pneumoniae*
MPP: *Mycoplasma pneumoniae* pneumonia
RMPP: Refractory *mycoplasma pneumoniae* pneumonia
GMPP: Genera *mycoplasma pneumoniae* pneumonia
WBC: White blood cell
ESR: Erythrocyte sedimentation rate
LDH: Lactate dehydrogenase
CRP: C-Reactive protein
AUC: Area under the curve
ROC: Receiver operating characteristic
CAP: Community-acquired pneumonia
PPD: Protein purified derivative
ECG: Electrocardiogram
N%: Proportion of neutrophils
L%: Proportion of lymphocytes
ALT: Alanine aminotransferase
AST: Aspartate aminotransferase
ULN: Upper limit of normal
